# Immunovirotherapy for Pediatric Solid Tumors: A Promising Treatment That is Becoming a Reality

**DOI:** 10.3389/fimmu.2022.866892

**Published:** 2022-04-13

**Authors:** Daniel de la Nava, Kadir Mert Selvi, Marta M. Alonso

**Affiliations:** ^1^Health Research Institute of Navarra (IdiSNA), Pamplona, Spain; ^2^Programs in Solid Tumors and Neuroscience, Foundation for the Applied Medical Research, Pamplona, Spain; ^3^Department of Pediatrics, Clínica Universidad de Navarra, Pamplona, Spain

**Keywords:** oncolytic viruses, pediatric solid tumors, pediatric brain tumors, sarcomas, neuroblastoma, clinical trials

## Abstract

Immunotherapy has seen tremendous strides in the last decade, acquiring a prominent position at the forefront of cancer treatment since it has been proven to be efficacious for a wide variety of tumors. Nevertheless, while immunotherapy has changed the paradigm of adult tumor treatment, this progress has not yet been translated to the pediatric solid tumor population. For this reason, alternative curative therapies are urgently needed for the most aggressive pediatric tumors. In recent years, oncolytic virotherapy has consolidated as a feasible strategy for cancer treatment, not only for its tumor-specific effects and safety profile but also for its capacity to trigger an antitumor immune response. This review will summarize the current status of immunovirotherapy to treat cancer, focusing on pediatric solid malignancies. We will revisit previous basic, translational, and clinical research and discuss advances in overcoming the existing barriers and limitations to translate this promising therapeutic as an every-day cancer treatment for the pediatric and young adult populations.

## Introduction of Pediatric Cancer

Pediatric cancer includes all malignancies that occur in children and adolescents between birth and 19 years of age, and it is estimated that every year approximately 400,000 cases are diagnosed worldwide (1, 2). Recent advances in cancer research have resulted in a marked increase in the cure rates of both adult and pediatric cancers. However, cancer remains a significant cause of death for children and adolescents (3). Among the factors that explain the improvement in survival rates are the optimization of supportive care, advances in biological and clinical tumor characterization and the development of new risk-adapted therapies are the most remarkable (4, 5).

Unfortunately, this improvement has not always been accompanied by improved quality of life because of side effects and long-term health complications in survivors of childhood cancers as they reach older age (6). These data underscore the necessity to develop safe and efficacious treatments that overcome the current limitations in the field of pediatric cancer.

In recent years, cancer therapy has experienced a remarkable transformation due to the advent of new classes of immunotherapies, including immune checkpoint inhibitors, bispecific T cell engagers (BiTEs) and CAR-T cells. In fact, CAR-T cells have provided a new paradigm for treatment, especially for liquid tumors (7, 8). Another approach that has gained popularity is the use of oncolytic viruses (OVs). These viruses combine their cytotoxic capacity with the ability to trigger an immune response, rendering them interesting therapeutic tools. The notion of viruses as anticancer agents came from anecdotic observations where tumors regressed spontaneously after the patients naturally acquired viral infections (9–11). These reports were mainly from the early 1900s, and it was not until the late 1980s that OVs were evaluated in depth, in part due to the development of research tools such as cell lines and animal models that have facilitated the evaluation of these agents (12). Since then, multiple investigations have been carried out, and the first OV has been approved for clinical use in the USA. The use of Talimogene Laherparepvec in clinical practice for recurrent melanoma represents a before-and-after picture for OVs, indicating the possibility of developing new, functional and perfectly designed tools for tumor treatment (13).

In this review, we discuss the role of OVs as a therapy for pediatric solid malignancies. We review the different types of viruses and their mechanism of action. We recapitulate the basic, translational and clinical research using virotherapy, alone or in combination with other therapies, to treat pediatric solid tumors, and we conclude with our thoughts regarding the potential future and hurdles for the development of this field to achieve its full therapeutic potential.

## Oncolytic Viruses

**Figure 1:**
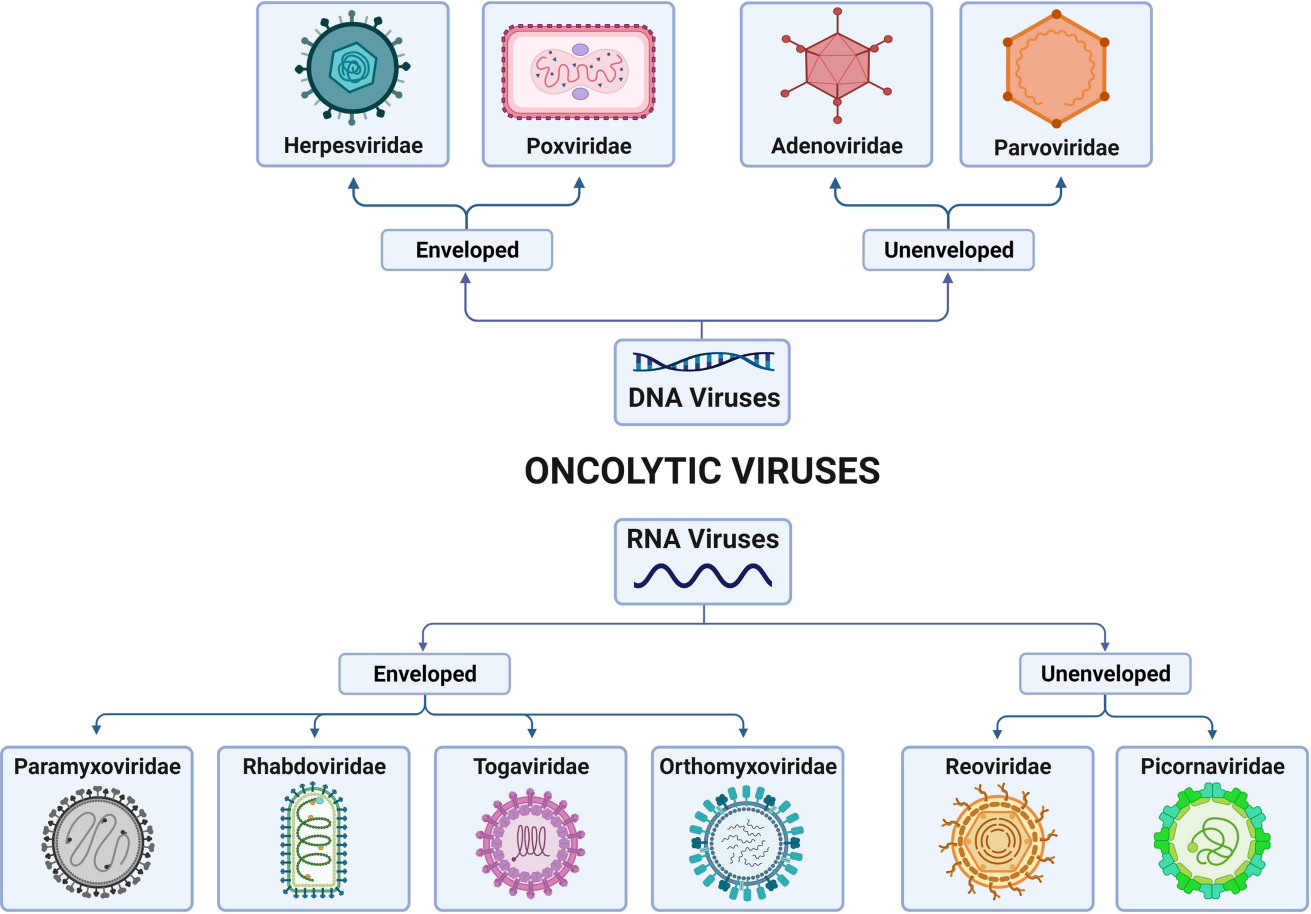
Classification of families of oncolytic viruses (OVs) according to the genome and presence/absence of the viral envelope. OVs can be differentiated into DNA viruses and RNA viruses, depending on their genomic nature. In addition, both types can present, or not present, virus envelopes. Thus, DNA-enveloped viruses include those of the Herpesviridae and Poxviridae families; non-DNA-enveloped viruses include those of the Adenoviridae and Paroviridae families; RNA-enveloped viruses include those of the Paramyxoviridae, Rhabdoviridae, Togaviridae and Orthoyxoviridae families; and non-RNA-enveloped viruses include those of the Reoviridae and Picornaviridae families. Created with BioRender.com.

Oncolytic viral therapy is a promising therapeutic method that employs naturally occurring or genetically modified OVs that selectively proliferate in and kill tumor cells while causing no harm to healthy cells (14). OVs can be classified as DNA or RNA viruses on the basis of their genome. Furthermore, they differ in their viral envelope based on host cell membranes and viral glycoproteins. According to these criteria, these viruses can be classified as enveloped DNA OVs (herpesvirus, poxvirus), unenveloped DNA OVs (adenovirus, parvovirus), enveloped RNA OVs (paramyxovirus, rhabdovirus, togavirus, orthomyxovirus), or unenveloped RNA OVs (reovirus, picornavirus) (**Figure 1**).

**Figure 2:**
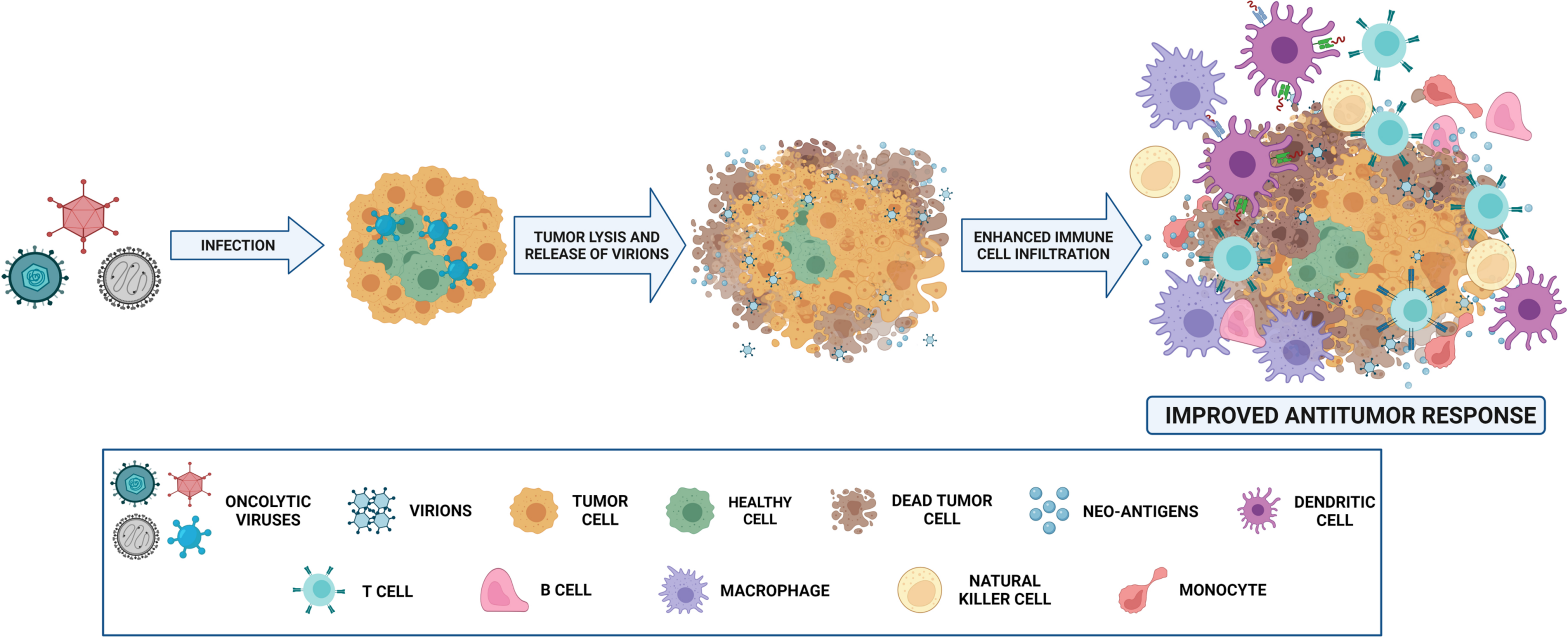
Mechanism of action of oncolytic viruses (OVs). OVs, administered intravenously or intratumorally, infect normal and tumor cells. Nevertheless, they replicate only in malignant cells, and virion release then occurs. The infection and destruction of tumor cells lead to the release of antigens that cause proinflammatory recruitment of different immune cells, such as T and B cells, macrophages, NK cells, monocytes and dendritic cells. Thus, antitumoral effect is mediated not only by oncolytic capability, but also by immune microenvironment reshaping. Created with BioRender.com.

OVs elicit antitumor responses mainly through two mechanisms: selective killing of tumor cells and stimulation of systemic antitumor immunity (15). Cancer cells provide an ideal setting for the selective replication of various OVs that take advantage of physiological changes in these cells. Several signaling pathways engaged in viral elimination, including interferon, Toll-like receptor (TLR) or Janus kinase-signal transducer and activator of transcription (JAK-STAT) pathways, may be defective or inhibited, enabling OVs to spread in tumor cells. Regarding infectivity, cancer cells may also overexpress different surface receptors, such as CD46, ICAM-1, CD55, CD155 or integrins, allowing OVs to infect cancer cells (16). In addition to directly killing infected tumor cells *via* several oncolytic mechanisms, OVs have the ability to turn tumors from immunologically ‘cold’ to ‘hot’ by inducing proinflammatory conditions within the tumor microenvironment (TME) (15). Oncolytic cell death and subsequent release of tumor-associated antigens can induce innate and adaptive immune responses, resulting in therapeutic responses in both locally injected tumors and tumor metastases (15, 17, 18) (**Figure 2**).

With their multimodal antitumor activity, oncolytic virotherapies are now a key subject of study in cancer treatment research, and many clinical trials are currently being conducted for the utilization of OVs as therapeutic agents for different types of advanced malignancies (19, 20). OVs for different types of malignancies are chosen based on a variety of factors. Some of them may have intrinsic tropism and a preference for selective replication in cancer cells, whereas others can be genetically modified to elicit selective targeting of cancer cells (21, 22). Generally, OVs infect tumor cells *via* specific receptors on the cell surface. Once inside, the virus particles migrate through the cytoskeleton and start replication, being internalized or not into the nucleus. The viral seizure of the cellular machinery allows the generation of new virions, which then facilitate lysis of the infected cell and spread to infect new cells (23). New advances in oncolytic virotherapies are being achieved with genetic deletions and genetic engineering to improve tumor-selective replication and oncolytic capability while lowering viral pathogenicity for patient safety (21, 22, 24). Additionally, OVs can be designed to express novel therapeutic genes to enhance the antitumor action, generation of immunological responses, and suppression of tumor angiogenesis, along with other mechanisms (21, 25). Reoviruses, parvovirus H-1 and Newcastle disease virus (NDV) are examples of OVs that are naturally inclined to replicate in cancer cells (20). On the other hand, other OVs have been genetically modified for oncoselectivity, including adenoviruses, vaccinia virus (VV), vesicular stomatitis virus (VSV) and herpes simplex virus (HSV) (26, 27).

## Preclinical Studies Using Virotherapy in Pediatric Solid Tumors

### Adenovirus

Adenoviruses are among the most commonly used OVs in research (28). Among the different pediatric solid tumors, those of the central nervous system (CNS) are the most common malignancies among children aged 0 to 14 years, even more so than leukemias, and are among the most prevalent and deadly within the adolescent group (5–24) and young adults (25-39 years) (29). There are more than 100 types of brain tumors (30–32), each with its own characteristics. However, for the purpose of this review, we will focus on those with poor prognosis.

One adenovirus that has been extensively characterized in pediatric and adult brain tumors is Delta-24-RGD. This adenovirus, serotype 5, was specifically designed to destroy tumor cells (33, 34). It contains two genetic modifications: a 24-base pair deletion in E1A, which restricts virus replication in tumor cells, and the addition of the RGD-4C binding motif, which improves the infectivity, allowing the virus to target tumor cells *via* avβ3 and avβ5 integrins, which are overexpressed in a wide range of tumors. Delta-24-RGD showed a robust antiglioma effect in preclinical and clinical studies in adult patients with recurrent gliomas (34–36). Moreover, the virus displayed the capacity to trigger immune-mediated responses with an increased number of immune populations inside the tumors (37, 38). Our group has evaluated this virus in the context of high-risk pediatric brain tumors. We observed that treatment with Delta-24-RGD resulted in increased survival in human xenograft and syngeneic mouse models of pediatric high-grade gliomas (pHGGs) and diffuse midline gliomas (DMGs) (39). In immunocompetent mice, virus treatment led to robust recruitment of lymphocyte populations (including CD4+ and CD8+). Delta-24-RGD combined with radiotherapy exerted an improved antitumor effect in this type of tumors (40). Furthermore, we evaluated this virus in models of atypical teratoid/rhabdoid tumors (AT/RTs) and CNS-primitive neuroectodermal tumors (CNS-PNETs), rare pediatric embryonal tumors with a survival time of 6-12 months (41, 42). The adenovirus replicated efficiently in AT/RT and CNS-PNET cell lines, leading to a robust cytotoxic effect. *In vivo*, Delta-24-RGD expanded the overall survival in several animal models, leading, in some cases, to a long-term survival rate of 70%. The interrogation of the immune response triggered by Delta-24-RGD in humanized immunocompetent mouse models revealed an increase in CD8+ T cell infiltration and a general remodeling of the TME toward a proinflammatory phenotype (43). In this line of thinking, another adenovirus, VCN-01, armed with hyaluronidase, which allows the degradation of the extracellular matrix (44), also significantly extended the overall survival of mice bearing orthotopic PNET (45). Mesenchymal stem cells (MSCs), as OV carriers, have also been studied in treating DMGs. Chastkofsky and colleagues encapsulated the adenovirus CRAd.S.pK7 into MSCs to facilitate its delivery to the brainstem and to avoid potential fast clearance by the immune system (46). Although the virus replicated *in vitro*, the experiments performed in animal models did not show clinical benefit, and it was necessary to add radiotherapy to extend the overall survival.

Another strategy used to enhance the efficacy of oncolytic adenoviruses has been combination with gene therapy. In this sense, Arnone et al. explored the idea of treating a pediatric high-grade glioma with the OV Delta-24 in combination with a replication incompetent adenovirus encoding a BiTE which targets human hepatocellular carcinoma A2 receptor (EphA2), a protein that is correlated with tumor aggressiveness and poor patient outcome. The authors showed that the combination treatment was more efficient than either treatment alone in improving tumor burden and overall survival (47).

Neuroblastoma is a rare neuroendocrine childhood cancer that arises in any neural crest element from the developing sympathetic nervous system (48). It is the most common extracranial solid tumor in childhood and the most common malignancy diagnosed during the first year of life (49). Although outcomes in these patients have improved in recent decades, this improvement is attributable mainly to better cure rates among patients with low-risk neuroblastoma (49), whereas children bearing the more aggressive form of the disease have shown a modest advance (50). In this context, the adenoviruses OBP-301 and OBP-702, the tumor specificity of which is driven by the hTERT promoter, have been evaluated. The authors showed that treatment with either of these viruses produced an antitumor response in cell lines with high hTERT expression and reduced growth in a subcutaneous neuroblastoma model (51). Other approaches have employed cellular carriers to deliver the virus such as Celyvir; autologous MSCs loaded with Icovir-5 (an adenovirus dependent on an aberrant RB pathway). Mice bearing neuroblastomas and treated intravenously with Celyvir displayed a reduced tumor volume, recruitment of immune cells and a weaker protumoral and stronger inflammatory profile in the TME (52). This strategy is currently being evaluated in the clinic and is discussed below.

Pediatric sarcomas, which account for 10% of solid tumors in children, are a group of mesenchymal tumors originating from bone or soft tissue precursors (53). Although current treatment regimens based on chemotherapy, surgery and radiation have improved the 5-year OSR to 60-70%, patients with metastatic disease or recurrence have a poor prognosis, with a 5-year OSR of 30% or less (54, 55). The lack of efficacy of emergent therapies such as immunotherapy (56) has prompted the emergence of OVs as an alternative solution.

Our group has evaluated the antisarcoma effect of the RB pathway-based viruses Delta-24-RGD (57) and VCN-01 (58). Both adenoviruses were able to control tumor volume, and specifically for Delta-24-RGD, the use of cisplatin as a combinatorial treatment improved the antitumor virus response, showing that combination therapies are, in fact, a possible solution. In the quest to further improve the efficacy, our group engineered D24-ACT, which is based on the D24-RGD platform and armed with the immune costimulatory molecule 4-1BB ligand (4-1BBL), to improve the antitumor immune response. Local treatment with Delta-24-ACT in mice bearing orthotopic osteosarcoma murine tumors led to a reduction in both the primary tumor and metastases, and a significant increase in CD3+ and CD8+ T cells, among other immune populations, was found when comparing D24-ACT vs. D24-RGD (59). Our results suggest that potentiating the immune response could boost the efficacy in this type of tumor. In another study, a murine version of Celyvir (OAd-MSCs) was tested in combination with granulocyte-colony stimulating factor (G-CSF). The combination significantly reduced tumor growth *in vivo*, with tumors presenting higher infiltration of some immune cell populations (including CD4+ and CD8+ T cells) and reduced T cell exhaustion (60). OBP-502 has also been evaluated in osteosarcoma preclinical models. This adenovirus reduced the viability of cancer cells and induced immunogenic cell death *in vitro*, whereas intratumoral injection in combination with an anti-PD-1 antibody in subcutaneous models reduced tumor growth and enhanced tumor-infiltrating CD8+ T cells (61).

### Herpes Simplex Virus

HSV is among the largest DNA viruses developed for gene transfer. It is nonintegrative, very potent as a lytic virus, highly replicative and with high cell tropism (62). HSV type I G207, which contains deletions in both copies of the neurovirulence gene γ_1_34.5 and a disabling lacZ insertion within the ICP6 gene (63), has been proven to be safe when injected into the cerebellum (64) and developing mouse brains (65). In preclinical studies, HSV-1 G207 and M002 (encoding human interleukin-12) demonstrated efficacy in pediatric high-grade glioma (66) and medulloblastoma (67). Another oncolytic herpes virus, HSV1716 (Seprehvir), showed efficacy in preclinical studies of high-grade gliomas and DMGs *via* changes in cytoskeletal dynamics and in molecular pathways related to cell polarity, migration, and movement (68). HSV-1 rRp450, which expresses the rat CYP2B1 enzyme and is able to activate the chemotherapeutic prodrug cyclophosphamide, prolonged overall survival in medulloblastoma and AT/RT, and its efficacy was further enhanced when cyclophosphamide was included in the treatment schedule (69).

In neuroblastoma models, HSV-1 M002 produced cell death in different neuroblastoma cell lines *in vitro* and reduced, alone and in combination with radiation, the tumor growth of this tumor *in vivo* (70). Similarly, a nestin-targeted oncolytic HSV also killed neuroblastoma tumor-initiating cells and prevented tumor formation in xenograft-bearing mice (71). FusOn-H2 (type 2 HSV), which specifically targets tumor cells with an aberrant Ras signaling pathway, exhibited efficacy in a syngeneic mouse model not only at the virus injection site but also at distant metastases (18).

In Ewing sarcoma, the second most common bone tumor in children and adolescents and a difficult to treat cancer (72), HSV-1 rRp450 was combined with macrophage reduction drugs. The combined treatment improved the efficacy of each agent alone and led to a reduction in M2-like macrophages in the tumor and spleen (73). HSV has also been tested in Rhabdomyosarcoma, the most common soft tissue sarcoma (74). Similar to other sarcomas, the chance of cure for metastatic and recurrent tumors is incredibly low (< 20%). In this context, HSV-1 M002 exhibited replication and oncolytic activity *via* apoptosis, reduced tumor growth and acted synergistically with radiation in subcutaneous mouse models (75). This virus has been additionally evaluated in serendipitous murine models of undifferentiated sarcoma, leading to an increase in effector CD4+ and CD8+ T cells, activated monocytes and a decrease in myeloid-derived suppressor cells (76).

### Other Oncolytic Viruses

Parvovirus H-1 (H-1PV) is an apathogenic in humans and non-recombinant OV that occurs naturally in rats (77), and long-term survival was observed for adult high-grade gliomas mouse models after intratumoral, intravenous or intranasal virus application (78, 79). H-1PV showed efficacy in *in vitro* models of pediatric high-grade glioma (80), medulloblastoma (81) and Ewing sarcoma but failed to improve survival *in vivo* in this tumor (82).

Medulloblastoma and pleomorphic xanthoastrocytoma (a rare condition comprising <1% of all primary brain tumors) have been evaluated with PVSRIPO. PVSRIPO is an attenuated polio: rhinovirus chimera without neurovirulence and had been evaluated previously in adult recurrent glioblastoma patients with promising results (83). The authors observed that PVSRIPO could be used against these two malignancies *in vitro* (84). Other types of OVs have been used in preclinical models, such as a measles virus in AT/RT models (85) or the picornavirus Seneca Valley virus (SVV-001) in pediatric glioma (86), with an improvement observed in mice bearing tumor cells. Another study described the use of the oncolytic vesicular stomatitis virus VSV^ΔM51^ and oncolytic myxoma virus (87) as treatments *in vitro* and *in vivo* with good responses, although the models used were subcutaneous.

A specific Semliki Forest virus (SFV4miRT), an *Alphavirus* belonging to the *Togaviridae* family and modified to reduce neurovirulence through insertion of three microRNAs, prolonged survival in neuroblastoma and glioblastoma mouse models with low interferon-α/β secretion (88). Another OV belonging to this family, Sindbis virus (SINV), exhibited efficacy *in vitro via* apoptosis, reduced tumor growth and extended overall survival in subcutaneous neuroblastoma models (89). An attenuated, nonneurovirulent poliovirus was evaluated and exhibited replication in neuroblastoma cells and significant reduction in tumor growth in subcutaneous tumor-bearing mice (90). Additionally, in another study, MV-CEA (an engineered measles virus) produced neuroblastoma cell death *via* apoptosis *in vitro* and extended the overall survival in xenograft models after five doses were injected intratumorally (91). Interestingly, treatment with an OV that expresses a CXCR4 antagonist injected intravenously augmented the efficacy of DC vaccines (92). Another oncolytic Seneca Valley virus (NTX-010) proved effective in a subcutaneous neuroblastoma and Ewing sarcoma models (93). Zika virus has been evaluated as an OV. Mazar and colleagues showed that neuroblastoma cells are widely permissive to Zika infection and require CD24, although the efficacy in cell death has not been proven (94).

Measles OV decreased tumor growth of subcutaneous xenografts and prolonged survival with orthotopic and pulmonary metastatic osteosarcoma tumors (95). Also in osteosarcoma, other viruses such as the previously mentioned parvovirus H-1PV (96), oncolytic vesicular stomatitis virus VSV^ΔM51^ in combination with phosphoinositide 3-kinase inhibitor (97) and myxoma virus treated with immune checkpoint inhibitors (98) successfully demonstrated an antitumor effect. Interestingly, coculture of an Ewing sarcoma cell line with NK cells led to a better oncolytic effect of a measles OV (99).

The reovirus Reolysin exhibited efficacy in the treatment of osteosarcoma, Ewing sarcoma and rhabdomyosarcoma cell lines *in vitro* and in flank xenografts *in vivo*, in combination with radiotherapy or the chemotherapeutic cisplatin, injecting three doses every three weeks *via* tail vein administration (100). Phelps and collaborators developed a recombinant oncolytic myxoma virus engineered with CRIPSR/Cas9 gene editing capability targeting *NRAS* (Myx : NRAS). While nonmodified myxoma virus slightly improved the overall survival in rhabdomyosarcoma subcutaneous models, the clinical effect was improved greatly by using Myx : NRAS, and long-term survival was achieved (101). VSV^ΔM51^ has also been evaluated in rhabdomyosarcoma and synergized with the Smac mimetic compound LCL161 *in vitro* and *in vivo* in a syngeneic murine model (102). In another elegant work, Petrov and colleagues used canine adipose-derived MSCs as carriers of vaccinia viruses as a “Trojan Horse” to circumvent an early immune attack to treat a canine soft tissue sarcoma (103).

## Oncolytic Viruses in Clinical Trials

In this section, we recapitulate the latest updates from clinical trials using OVs as therapeutic agents in pediatric solid cancers. All clinical trials using OVs that have been conducted in pediatric populations are included in **Table 1**.

**Table 1:** Clinical trials, completed or active, using oncolytic viruses as treatment in pediatric solid tumors.

Family	Name	Phase/Country	Modifications	Target Disease	Route	Identifier/Reference
Adenovirus	Delta-24-RGD/ DNX-2401	I/Spain	24-base pair deletion in the Rb-binding region of the E1A gene, insertion of an integrin-binding motif RGD	DMG	intratumoral	NCT03178032 (104)
	VCN-01	I/Spain	24-base pair deletion in the Rb-binding region of the E1A gene, insertion of an integrin-binding motif RGD, human hyalurodinase gene insertion	Refractory retinoblastoma	intravitreal	NCT03284268
	Icovir-5 (Celyvir)^a^	I/Spain	24-base pair deletion in the Rb-binding region of the E1A gene, integrin-binding motif RGD insertion, E2F-1 promoter insertion	Metastatic/Refractory solid tumors	intravenously	NCT01844661 (105)
	Icovir-5 (AloCELYVIR)^b^	Ib/Spain		DMG/ Medullablastoma	intravenously	NCT04758533
Herpex Simples Virus Type 1	HSV1716/Seprehvir	I/USA	Gene encoding ICP 34.5 protein (RL1) deletion	Non-CNS solid tumors	Intratumoral /intravenously	NCT00931931 (106)
	G207	I/USA	Deletion of the diploid γ134.5 gene, viral ribonucleotide reductase (UL39) disruption by lacZ insertion	Recurrent/Refractory cerebellar brain tumors	intratumoral	NCT03911388
	G207	I/USA		Progressive/Recurrent supratentorial brain tumors	intratumoral	NCT02457845 (107)
	G207	I/USA		Recurrent/Progressive high-grade gliomas	intratumoral	NCT04482933
Vaccina Virus	JX-594	I/USA	Thymidine kinase gene (TK) disruption, human GM-CSF and β-galactosidase gene insertion	Refractory solid tumors	intratumoral	NCT01169584 (108)
Reovirus	Reolysin	II/USA	Unmodified	Metastatic sarcomas	intravenously	NCT00503295
	Reolysin	I/USA and Canada		Relapsed/Refractory Solid Tumors	intravenously	NCT01240538 (109)
Picornavirus	Seneca Valley Virus	I/USA	Naturally occurring	Advanced Solid Tumors with Neuroendocrine Features	intravenously	NCT01048892 (110)
Poliovirus/ rhinovirus chimera	PVSRIPO	Ib/USA	Poliovirus type I containing heterologous internal ribosomal entry site of human rhinovirus type 2	Recurrent malignant glioma (Grade III or IV)	intratumoral	NCT03043391

Updated Jan 2022. ^a^Celyvir, Celyvir system consists on autologous MSCs carrying ICOVIR-5. ^b^AloCELYVIR, AloCELYVIR system consists on allogenic MSCs carrying ICOVIR-5.

One of the first clinical approaches using an OV in the pediatric population was a case report published in 2006 (111). A 12-year-old boy with anaplastic astrocytoma who was subjected to conventional therapy (surgery, radiation and chemotherapy) and progressed was treated with MTH-68/H (attenuated strain of paramyxovirus NDV) as a compassionate use. MRI scans showed 30% tumor regression two months after viral infusion. However, the patient’s condition began to decline 4 months after the first MTH-68/H treatment due to the growth of new tumor nodules, and additional surgery was required. The patient passed away after 41 months.

Since then, several formal clinical trials utilizing different OVs have been performed, all of them looking into safety and feasibility. For example, Seneca Valley virus (NTX-010) was used in advanced solid tumors with neuroendocrine features in a phase I trial that included adults and children (112). Patients with neuroblastoma, rhabdomyosarcoma, Wilms tumor, carcinoid tumor and adrenocorticocarcinoma were included in this trial, which included two arms: one with just the OV and another with the virus combined with cyclophosphamide. Dose-limiting toxicity was observed in the first arm but not in the second. Adverse events of grade ≤3, such as leukopenia, neutropenia, nausea, or anemia, were described. Unfortunately, no complete or partial responses were observed, and only stable disease was observed in 6 out of 12 patients in the first arm (neuroblastoma n = 4, carcinoid tumor n=1; and rhabdomyosarcoma n=1) and 4 out of 6 in the second arm (neuroblastoma n = 2; and Wilms tumor n = 2).

JX-594 (Pexa-Vec; a vaccinia virus) was evaluated in six patients with metastatic neuroblastoma, hepatocellular carcinoma and Ewing sarcoma. No severe toxicity was associated with virus administration. Regarding efficacy, 4 out of 6 patients presented stable disease and 2 progressive disease, and uninjected lesions progressed in all patients except one, whose lung nodules were stable (108).

The oncolytic reovirus Reolysin was the drug chosen for another phase I clinical trial (109). Twenty-four children with relapsed or refractory extracranial solid tumors were administered Reolysin intravenously for 5 consecutive days every 28 days, alone or in combination with the chemotherapeutic cyclophosphamide. Adverse events of grade ≤4 and even grade 5 thromboembolism in one patient were described. Regarding efficacy, only three patients with stable disease received a second cycle, whereas two patients received a third cycle prior to progressive disease.

In a study published by Melen and collaborators, Celyvir was chosen for compassionate use for 13 patients with advanced refractory neuroblastoma. Children received weekly multidoses as a sole treatment. The only adverse effects found were mild and autolimited viral-related toxicities, and none of the patients experienced grade 3+ toxicities. Regarding clinical outcomes, progression was the most common (n=8), with stable disease (n=1), partial response (n=3) and complete response (n=1). The authors found that the nonresponder patients’ MSCs showed lower levels of expression of adhesion molecules and migration capacities, and a higher number of T cell lymphocytes was found in responder patients (113).

Our group has finished a phase I, dose-escalation clinical trial using DNX-2401 followed by standard radiotherapy in naïve DMGs, in which 12 patients were enrolled (114). Correct infusion of viral particles was checked using gadolinium in all patients (104). The treatment regimen was well tolerated, with asthenia, headache, vomiting, pyrexia and neurological deterioration being the most commonly reported adverse events. The three serious adverse events reported were grade 3 abdominal pain, grade 3 lymphopenia and grade 3 clinical deterioration. Regarding efficacy, tumor reduction was observed in 9 out of 12 patients. The final report of this study is still pending. The preclinical studies mentioned above have allowed the transfer of HSV-1 G207 in a phase I clinical trial to treat pediatric high-grade gliomas, in which supratentorial tumors had, at recurrence, a median life expectancy of only 5.6 months. The injection was performed *via* catheterization, and dose-limiting toxicities in the 12 patients enrolled were classified as grade 1 and 2 (more common) and 3 (less common) related to HSV-1 G207. The median overall survival was 12.2 months, and 4 patients were still alive 18 months after HSV-1 G207 injection. Importantly, posttreatment tissues from patients showed a substantial increase in CD3+, CD4+ and CD8+ T cells (107).

The oncolytic herpes virus HSV1716, mentioned above in preclinical research, has been evaluated in a preclinical trial for the pediatric population (115). In this study, Streby et al. recruited nine patients with relapsed or refractory extracranial solid tumors (pediatric sarcomas, neuroblastoma and cholangiocarcinoma). No dose-limiting toxicities were observed, and all the adverse events were of grade ≤ 3. However, only two patients exhibited disease stabilization in response to the virus, and months later, the tumors started to progress (106).

## Perspective and Concluding Remarks

As depicted along this review, OVs have exhibited potential applications in the treatment of pediatric solid tumors with encouraging results. However, OVs have been regularly evaluated in tumors whose survival has not improved much in recent decades (including pediatric cancers). Despite the great efforts and multitude of clinical trials carried out, these diseases continue to be devastating. This constitutes a double-edged sword for OVs. On the one hand, they offer an alternative for cancer patients for whom the currently available treatments do not lead to sufficient improvement, much less cure, of the pathology. On the other hand, all of these tumors are very aggressive, and there could be no room to obtain tangible improvement, at least with monotherapy. Notably, although OVs have shown marked antitumor effects in preclinical models, their clinical translation has not been so successful. Indeed, most of the clinical trials recapitulated the above recruitment of patients in their last stages of their diseases and therefore with modest results. Nevertheless, in recent years, combinations of OVs with several chemo- and immunotherapy regimens have been proposed, and preclinical research and clinical trials are currently being conducted that show benefit compared to OVs alone (116–119).

Another point in favor of OVs is their low toxicity and extremely safe profile with very few secondary effects. This makes them very attractive, especially in the pediatric population. Viral production is also not a problem, as these viruses are easy and affordable to manufacture.

We consider that the main disadvantage of OVs is the route of administration. In intravascular administration, OVs are recognized and inactivated by humoral components of the innate and adaptative immune system in the blood (120), so this route is frequently dismissed. Therefore, intratumoral delivery is the dominant route, which allows direct targeting of the tumor using simple clinical procedures. However, there are also some difficulties, such as the presence of tissue barriers that might prevent the spread of the virus or the existence of metastases which might compromise the oncolytic efficacy. For that reason, approaches that improve virus delivery are under investigation and will be key to the further development of the field. In that regard, the use of cellular carriers such as MSCs (as explained above) (46, 52, 60, 103, 113), protective coatings and genetic modifications of OVs are other strategies that are considered for delivery optimization (121).

Interpretation of virotherapy responses through imaging within the clinical trial is another cornerstone where numerous efforts are being allocated. It is imperative to understand better what the imaging is telling us and define parameters that allow us to identify responses and other biological parameters intrinsic to the treatment with OVs.

In closing, the establishment of OVs as a therapeutic option for the treatment of tumors with poor prognosis, including pediatric solid malignancies, is encouraging. In recent years, investigations using OVs as therapy have grown exponentially. The FDA approval of Talimogene Laherparepvec has demonstrated that OVs are actually being considered as therapeutic options. Although we still need to overcome some barriers regarding OV application, their feasibility and, on some occasions, efficacy in treatment have been demonstrated. Further efforts will be needed and, given that virotherapy is now in its adolescence, there is great room for optimization. In the short-middle term, we believe that OVs will constitute a feasible therapeutic option to use alone or in combination with other strategies, for patients with pediatric solid tumors.

## Author Contributions

DN: Conceptualization, writing, review, editing and figure design. KS: Writing and figure design. MA: Conceptualization, supervision, writing, review and editing. All authors contributed to the article and approved the submitted version.

## Funding

The performed work was supported through a Predoctoral Fellowship from Instituto de Salud Carlos III (FI20/00020 DN), Instituto de Salud Carlos III y Fondos Feder (PI19/01896 MA); Fundación La Caixa (MA); Fundación El sueño de Vicky, Asociación Pablo Ugarte-Fuerza Julen, Fundación ADEY, Fundación ACS, (MA); Department of Defense (DOD) Team Science Award undergrant (CA 160525 MA); ChadTough Defeat DIPG Foundation (MA) and AECC (PRYGN21937ALON MA). This project also received funding from the European Research Council (ERC) under the European Union´s Horizon 2020 Research and Innovation Programme (817884 ViroPedTher to MA).

## Conflict of Interest

MA has a research grant from DNAtrix not related to this work.

The remaining authors declare that the research was conducted in the absence of any commercial or financial relationships that could be construed as a potential conflict of interest.

## Publisher’s Note

All claims expressed in this article are solely those of the authors and do not necessarily represent those of their affiliated organizations, or those of the publisher, the editors and the reviewers. Any product that may be evaluated in this article, or claim that may be made by its manufacturer, is not guaranteed or endorsed by the publisher.
